# Food composition databases in the era of Big Data: Vegetable oils as a case study

**DOI:** 10.3389/fnut.2022.1052934

**Published:** 2023-01-05

**Authors:** Henrique Ferraz de Arruda, Alberto Aleta, Yamir Moreno

**Affiliations:** ^1^ISI Foundation, Turin, Italy; ^2^CENTAI Institute, Turin, Italy; ^3^Institute for Biocomputation and Physics of Complex Systems (BIFI), University of Zaragoza, Zaragoza, Spain; ^4^Department of Theoretical Physics, Faculty of Sciences, University of Zaragoza, Zaragoza, Spain

**Keywords:** food composition database, food composition, food nutrient, vegetable oils, healthy nutrition, food guidelines

## Abstract

Understanding the population's dietary patterns and their impacts on health requires many different sources of information. The development of reliable food composition databases is a key step in this pursuit. With them, nutrition and health care professionals can provide better public health advice and guide society toward achieving a better and healthier life. Unfortunately, these databases are full of caveats. Focusing on the specific case of vegetable oils, we analyzed the possible obsolescence of the information and the differences or inconsistencies among databases. We show that in many cases, the information is limited, incompletely documented, old or unreliable. More importantly, despite the many efforts carried out in the last decades, there is still much work to be done. As such, institutions should develop long-standing programs that can ensure the quality of the information on what we eat in the long term. In the face of climate change and complex societal challenges in an interconnected world, the full diversity of the food system needs to be recognized and more efforts should be put toward achieving a data-driven food system.

## 1. Introduction

In their seminal book published in 1940, McCance and Widdowson started by stating that: “*The nutritional and dietetic treatment of disease, as well as research into problems of human nutrition, demands an exact knowledge of the chemical composition of food”* (1). In the years to follow, researchers from all over the world—together with private companies and non-profit organizations—delved into the basic constituents of food in an effort to understand what we eat and how it affects us. However, despite the many advances, this task may be far from being complete.

The information on the composition of food items used to be compiled in Food Composition Tables (FCTs), although nowadays, many countries have updated them into Food Composition Data Bases/Data Banks (FCDBs). For instance, the EuroFIR project hosts the databases from 30 different countries. This information may come from four different sources: primary publications, secondary publications, unpublished reports, and analytical reports (2). In the first, the composition is extracted directly from journal articles. In the second, food information is compiled from other sources, which include reviews, books, reports, or other FCDBs/FCTs. The third source includes reports that are not publicly available, which include internal use reports. Lastly, analytical reports can be divided into two subcategories, namely specific and non-specific. The specific analytical reports are created to feed a particular FCDB/FCT, and the non-specific analytical reports contain data not obtained for this aim. The diversity of sources, analytical methods, variations of the same food, terminologies, and economic cost makes the procedure of collecting and integrating all this information a huge challenge.

The problem with these data is further exacerbated by the different criteria used in each country to create them, which can be partially explained by the diverse object pursued by each project. At least since 1982, there have been several initatives to harmonize procedures for better data comparability and interchange, such as INFOODS from FAO or EUROFOODS from COST (3). Even so, studies from the late 90s showed that mean intakes of individual nutrients for the same diet could vary up to 20–45% depending on the database used to estimate food composition (4). These differences were associated with systematic and random errors, variations in naming, terminology, or calculation procedures across databases, and to the intrinsic differences of food items in different countries. Many initiatives aimed at solving these problems either failed or succeeded only for a brief period but then aged badly due to lack of funding or a driving force (5).

In 1989, the United Nations published the INFOODS data interchange handbook with guidelines to improve data on the nutrient composition of foods, as they observed that such “data do not exist or are incomplete, incompatible, and inaccessible” (6, 7). In the European context, a clear indicator that this problem still needs to be solved is the number of initiatives that periodically appear to address these issues. From 1985 the NORFOODS group practiced data interchange among Nordic countries. Even though pioneering, it had some limitations. For instance, the data exchange was restricted to the data contained in the databases, but not the metadata (8). From 1995 to 1999, the EUROFOODS project created a working group to address the issues of food composition data management and interchange. The project led to a set of recommendations to make national databases compatible and facilitate data interchange (9). Independently, in 1990 the European Prospective Investigation into Cancer and Nutrition (EPIC) started. Its objective was to investigate the relationships between diet, nutritional status, lifestyle, and environmental factors and the incidence of cancer and other chronic diseases. Between 1992 and 1999, their participants collected data on the food intake of a large prospective cohort of over half a million individuals. The next step was to use FCDBs to estimate the nutritional intake. By that time, it was already known that FCDBs could be a significant source of imprecisions in this estimation (10). For this reason, in 1999, researchers reviewed the FCDBs available in the participant countries. They determined that: (i) the FCTs of different countries were not comparable due to the lack of reporting standards; (ii) comparisons within tables were problematic due to the use of very different sources; (iii) some tables were compiled with outdated information; (iv) there were inconsistent values of several nutrients across tables. As such, they established the necessity of creating standardized food composition tables for the countries involved in EPIC (11). This led to the creation of the Epic Nutrient Database (ENDB) in 2007, which had information for 26 components of 550–1,500 foods in 10 countries (12).

Concurrently, from 2005 to 2009, the European Commission sponsored the EuroFIR project, whose aim was to develop a pan-European system on food information (13). In 2010, the project was extended for 2 years with funding from the European Community's Seventh Framework Program under the name EuroFIR Nexus. It later evolved into a financially independent Association based in Brussels with the mission of maintaining high quality, validated national food composition data. An analysis carried out in 2021 showed that the documentation of the 26 European datasets included in EuroFIR was successfully standardized. Yet, full comparability of the datasets was not guaranteed as there were still many differences and inconsistencies. For instance, 15 out of the 26 datasets reported energy values calculated by factored summation with up to five different methods, while the others reported that the method was unknown or measured through analytical procedures (14).

In 2009, the European Food Safety Authority launched a pan-European food consumption survey - the EU Menu project (15). To create the methodological guidelines, it was necessary to obtain certain information on the nutritional composition of food. To do so, they funded the “Updated food composition database for nutrient intake project.” This project, finished in 2013, compiled information from 14 national food composition databases for almost 1750 food products. National datasets that did not contain all the information borrowed it from the datasets of other countries that, in the opinion of each compiler, consumed similar types of food. The percentage of values borrowed for each dataset ranged from 5 to 90%. Even extensive datasets borrowed 40% of the values as they were required to provide data for all elements included in the EFSA food list. It was presumed that most of the borrowed values belonged to seldom consumed foods in the country, and thus this should not have an important impact on the final intake estimation (16).

In 2018, the European Commission funded the Stance4Health project, in which one of the main tasks was to build a nutritional database to complete the national FCDBs from the countries involved in the project (Germany, Spain, and Greece) with as many foods and nutrients as possible. To add information for nutrients and biocompounds not present in those databases, they complemented the information with the FCDBs of Italy, the Netherlands and the United Kingdom, together with the FAO, USDA, and Phenol-Explorer databases. This way, the total contribution to the unified database of each of the three original sources of data was 15 9 and 2% for Germany, Spain, and Greece. Excluding polyphenols and focusing only on the 40 nutrients most commonly used in epidemiology, Spain and Germany had information about 88% of the nutrients and Greece 40% (17).

It is thus clear that, despite the huge advances produced in the last 40 years, the creation of a unified, reliable, and comprehensive database on food composition at the European level is far from complete. Globally, the situation is not better. Even though it is crucial to have regional and country-specific FCDBs, many countries do not have a national FCDB, and many of the ones that do are outdated, do not follow international standards in terms of quality, coverage, accessibility, and documentation. Furthermore, most of them, including European ones, contain < 25% of analytical data, and these data are usually old and not generated specifically for the FCDB (18). The situation is particularly challenging in Africa, where micronutrient deficiencies are one of the major public health challenges, and the data gaps on what people are eating (and their contents) make it very difficult to devise optimal strategies to improve diets and malnutrition (19).

The main contribution of this paper is to depict and exemplify the main limitations of the FCDBs. In particular, we quantify the differences in terms of the number of nutrients and compounds found in these databases. Further, we explore the age of the information contained in them, which is a problem often overlooked. Next, we examine the differences in terms of the compositions of various vegetable oils in a given FCDB and the discrepancies between FCDBs for the same oil. Although differences might be expected, we highlight inconsistencies that can be found even within the same database. Finally, we discuss how the development of current machine learning methods could help solve the problems identified in FCDBs.

## 2. The problems of current FCDBs

Currently available datasets have important limitations, including incomplete and outdated data, and not enough documentation on their sources and assumptions (18, 20). The nutrient content of food changes over time as a function of very complex processes involving many different aspects, from agricultural practices to policy and consumer pressure, and this is seldom reflected in them (21). Besides, it is common practice to complete missing elements with values obtained from other FCDBs or from the general literature. This might be reasonable for food items produced in few countries and traded globally, but it is problematic for locally produced food (22–24).

These errors may then propagate to other datasets and studies that might not be aware of those borrowings. Furthermore, research data, even if they are of sound analytical quality, may be biased in the selection of foods. Many FCDBs also do not include fortified foods, branded foods or a proper representation of the biodiversity of the food chain, which may lead to systematic errors in intake estimation (18). Besides the problems directly related to human nutrition and epidemiology, data gaps also have significant consequences for other surrounding areas, such as the sustainability of food systems and the pursuit of Sustainable Development Goals (SDGs).

In the following, we describe some of the most important problems of FCDBs, while in Section 3 we provide the analysis for the particular case of vegetable oils:

### 2.1. Missing values

While many countries possess their own FCDBs, the majority of them contain outdated and incomplete information. For instance, in 2021, it was determined that in the Dutch database (NEVO) about 50% of the items were missing information on the amount of vitamin K, hindering the assessment of the portion of the population with an adequate intake (25). This could be explained by the relatively recent discovery of the precise function of vitamin K in the 1970s (26), but at the same time shows the complexity of interpreting missing values. In fact, it is not always clear if there is a distinction between missing data and a value of zero for certain nutrients (17, 25). Thus, it is not possible to be sure if the compound is present or not in the food item, which may lead to important underestimations of nutritional intake.

### 2.2. Lack of sources

The lack of proper documentation of these databases may also hide important issues. Despite recent efforts, many databases are still missing their source of information, or the references might be incomplete (27). In the ones that report their sources, one can see that the information is extracted from the literature without taking into account important regional differences. To exemplify the problem, a literature review constrained to food produced and marketed in Brazil showed that the reported values were compatible with the ones contained in their national database for 81% of the products, decaying to 37.5% when comparing it with the database from the United States (USDA) (28).

Similarly, a comparison between the ENDB database (used in the European project EPIC) and the USDA found a strong agreement for macronutrients, but a weak agreement for starch, vitamin D and E, and thiamine-14% of the 28 compounds common in both datasets (24). This could be read as a sign of the small differences between the food composition of these two regions, at least in terms of macronutrients but, as we will see in Section 3.2 for the particular case of vegetable oils, many national databases extract their information from a common source, rather than by direct analysis. As such, when two databases report similar values, unless their source is stated, it is not possible to determine if that is because the food is similar in both regions or if they simply share the source.

### 2.3. Food fortification

In 2021, the United Kingdom joined the group of over 80 countries in which folic acid fortification of staples is mandatory in an effort to reduce the risk of neural tube defects in babies (29). Food fortification is becoming especially important in low- to middle-income countries, where micronutrient deficiencies are a widespread problem (30). In 2022, a study carried out in the Netherlands showed that up to 75% of the population consumed voluntarily fortified foods, resulting in a 64% higher intake of habitual micronutrients compared to non-users (31). Importantly, the study used the values reported on the labels of the products as an indicator of their composition. However, a Dutch study from 2017 showed that the vitamin D of some selected products ranged from 50 to 153% compared to the declared values (32). Similar results have been reported in the US (33), which could be related to the overages of vitamins added by producers to account for shelf life. Thus, using the reported values might produce under- or over-estimation of micronutrient intake in the population.

If, instead, one uses the information contained in FCDBs the problem might be even worse. The lack of information on the source, the outdated values and the important regional differences in terms of fortification policies may severely impact any estimations. For instance, Nordic countries have mild iodine deficiency and their fortification practices vary. A study from 2016 carried out by the NORFOODS project found out that the national animal feeding practices could produce two-fold differences in the iodine content of milk and eggs (34). As such, even for neighboring countries, the use of borrowed values for certain products might severely impact the estimations of nutrient intake and, hence, give rise to misguided policies. Thus, if it is necessary, the insertion of borrowed data in FCDBs shall be done only by experts adequately trained to understand the local nuances of the food and region.

In terms of coverage, even in countries with mandatory food fortification programs, data is not routinely collected (35, 36). For voluntarily fortified foods, given that they depend mainly on their producer, more information can be obtained in databases of branded foods. However, even though that, in Europe, declaring nutrients added for fortification purposes is mandatory, the information is not always clear. For instance, the authors of the Dutch branded food database (LEDA) could not determine the coverage of data on fortified nutrients due to unclear food name, ingredient descriptions and missing nutrient values (37).

### 2.4. Nutritional dark matter

Borrowing the term from genetics, nutritional “dark matter” refers to all those dietary factors that can influence our health but that remain largely invisible (38). For instance, it was recently shown that microRNAs present in plant foods can influence the genetic expression of enteric bacteria (39). There are thousands of biochemical compounds present in our food, but FCDBs were built to study only the nutrients that are essential for life. Due to the lack of data, nutritional epidemiology has focused on these few dozens of nutrients, disregarding elements such as amino acids and biogenic amines (40). While the USDA reports information on about 150 nutritional components present in food, FooDB, a large database on the chemical composition of food, contains more than 70,000 distinct biochemical compounds as of June 2022 (41). Yet, only 5% of them have been quantified. All this chemical diversity that remains invisible in common epidemiology may have an important effect on our health (42). Numerous initiatives are trying to compile this information from validated peer-review sources, such as Phenol-Explorer (43), but the current lack of harmonization introduces important challenges (44).

### 2.5. Branded foods

National FCDBs usually only document generic, non-branded foods. There are commercial databases that may provide this information, but they tend to be expensive and contain only details on macronutrients. In the European Union, pre-packaged foods must display their amount of some selected nutrients, but it is hard to validate their accuracy (45). Reformulation of processed foods is frequent as manufacturers try to keep their market share, increase their profits, make the food healthier, or are even forced to change due to government policies or consumer pressure. A study on the pizzas offered on the website of six supermarkets in the United Kingdom showed that, out of 903 pizzas, 10.8% of them changed their composition over 6 months and that 29.9% of them were either discontinued or new market entries (46). This information is hardly captured in most studies and, if it is, it might be restricted to the few nutrients reported in the labels of the product. Furthermore, many companies may rely on national FCDBs to estimate the nutritional value of their products rather than using direct measurements. If the limitations of the data are not clear, errors may propagate throughout the whole chain (21).

### 2.6. Outdated and misdated information

Even for raw products, the nutritional composition changes over time as a consequence of genetic selection, changes in agricultural practices or feed ingredients for farmed animals (47, 48). If FCDBs are not routinely updated, they may easily become obsolete (49). And, if they are, they should properly document all the changes so that one is aware if they were produced because the composition of food has changed or due to the improvement of the analytical techniques. Otherwise, for research studies over extended periods, variations in nutrient intake may reflect changes in the data rather than in the dietary patterns of the population (50). Similarly, dietary surveys must be analyzed with FCDBs compiled in the same period, or one risks finding spurious patterns due to the expected composition changes. Many institutions invest significant efforts in keeping the information updated, but this is not homogeneous. For instance, as we will see in Section 3.2, while the Spanish database has not been updated since 2010, the Danish database updated its information on coconut oil in 2022. More broadly, in a survey performed in 2019, researchers found that only 30 out of 107 available FCDBs had been updated in the previous 5 years (51).

### 2.7. Biodiversity

The differences in nutrient composition among varieties of the same product can be as important as between different species. For instance, an orange-fleshed banana from Micronesia can have 50 times more vitamin A than the common white-fleshed bananas, representing the border between nutrient deficiencies and nutrient adequacy (52). This biodiversity is seldom acknowledged, and general FCDBs usually report the information of a single sample or a naive average over different varieties of the same product. Over 15 years ago, FAO recognized the importance of biodiversity in nutrition and launched an initiative to create a database on biodiversity which could mitigate this lack of information (53, 54). Yet, despite the great advances produced by this initiative, and the relatively large size of the database, many common food items are not well-characterized yet (55). For instance, in the latest version of the food composition database for biodiversity, published in 2017, there is no information about olives, coconuts, palm or soybeans (56).

### 2.8. Climate change

Even though it is still early, research so far depicts a very complex picture in which some crops might benefit from higher temperatures—thanks to warmer temperatures—while others, specially those that require vernalization, will suffer (57). At the same time, faster growth might result in lower quality products both in terms of external appearance and internal composition (58). Changes in CO_2_ concentration may also have an impact on nutritional composition (59, 60). Furthermore, besides the changes directly produced by climate change, it may also be necessary to select and adapt crops to the new environmental conditions (51). Maintaining updated FCDBs will be a key element in devising the sustainable food supply of the future. And, at the same time, FCDBs can be a great resource for monitoring biodiversity and climate impacts in food systems.

## 3. Composition of vegetable oils in selected FCDBs

Although everyday there are more FCDBs in electronic format, accessing certain FCDBs can be complicated since some of them are not free, others are in analogical formats, and they may even lack an English translation (61). For those in digital format, in comparing several foods and nutrients, it can also be challenging to query automatically, and one needs to resort to manual exploration. Since this is a quite demanding process, and given their importance in the total caloric intake of the population, for this analysis, we focus on the particular case of vegetable oils as a case study.

To provide an overview of the current state of FCDBs, we have selected six databases covering several regions of the world:

- BEDCA: Is the Spanish FCDB, developed in 2010 as part of the EuroFIR project and has not been updated since then (62). It was compiled using the indirect method, that is, collecting all the information from different sources. Thus, it may not reflect the regional variability of certain products. It reports the source of information, but many references are empty. It does not contain information on fortified foods, which may impact the estimation of micronutrient intakes (63). Due to its weaknesses, Spanish commercial nutritional programs use a variety of other FCDBs (20). A study from San Mauro Martín and Hernández Rodríguez (64) studied the nutritional composition of the same diet estimated using different Spanish commercial nutritional programs. They showed that the estimated intake for each nutrient was highly heterogeneous, with differences in the range of 8–84% depending on the program.
- FRIDA: Is the Danish FCDB maintained by the National Food Institute (Technical University of Denmark) (65). It is easily accessible, updated frequently, and well-documented. It is composed by a mixture of direct analysis, information provided by several danish stakeholders and indirect information extracted from the scientific literature (66).
- USDA (Food Data Central): Is the FCDB from the United States Department of Agriculture (67). It is composed of five different databases, of which the Foundation Foods is the newest and most advanced. Until 2018 the main database was the SR Legacy, and it has been regarded for many years as a gold standard in the field, up to the point that many FCDBs in the world extracted their information from it. It is composed of data obtained from direct analysis, calculations as well as extracted from published literature.
- TBCA: Is the Brazilian FCDB (68). It is easily accessible and well-documented, although it lacks English translation. It contains an extensive selection of local products and their biodiversity, including many varieties for the same product. These products are mostly directly analyzed in Brazilian institutions, while for common foods in the world it comes from international databases such as the USDA.
- NIGERIA: The FCDB from Nigeria is small in terms of products but has an extensive selection of the most commonly used in the country (69). The documentation is scarce, although it reports the source of information for each product. Yet, they are not linked to each individual nutrient, and thus where each value comes from is unclear. The information is mostly extracted from published literature from Nigerian institutes—specially for local products—but also contains information from global sources such as the USDA.
- SMILING: The SMILING project aims at reducing micronutrient deficiency among children and women in South East Asia (Indonesia, Thailand, Cambodia, and Vietnam) (70). To create optimal diets for those countries, the first step was to compile regional FCDBs with information about the most commonly consumed products in the area, which they did in 2018. Due to the limited resources, they had to resort to indirect compilation. For several micronutrients they were not able to obtain local information and had to use international FCDBs. Besides, they realized that some of the sources they used were quite old and might have copied their values from non-regional sources. Thus, they claim, there is an urgent need to produce high quality data for local foods in the region (71). Note that many of these limitations are also present to some extent in databases of highly developed countries.

It must be noted that these databases were created through very different projects and budgets. For instance, BEDCA is the result of a project to build the first Spanish database using the EuroFIR standards. The project started in 2004 and finished in 2010, and thus it has not been updated ever since (20). In contrast, Food Data Central is a platform hosted by the USDA, a federal agency from the United States that has been analyzing foods and conducting human nutrition research for over 100 years (72).

We also complement the previous set with two other databases:

- FooDB: It is an online database that aims to be the largest resource on food constituents. It is easily accessible and well-documented, and reports thousands of chemical compounds with each food item. Unfortunately, most of them are not quantified, so the actual amount of reported nutrients is similar to national FCDBs. The information is extracted from other FCDBs as well as from public databases on phenols or pathways. Besides, the main source of information on nutrients are the USDA and FRIDA databases, and thus lacks information on regional biodiversity (41).
- EuroFIR: As previously described, the EuroFIR database is the result of the original EuroFIR project, which intended to create a homogeneous database for Europe. In contrast to the other databases, to access EuroFIR data, it is necessary to purchase a membership, which was imposed to assure the long-term sustainability of the initiative (73). The EuroFIR guidelines are one of the standards used in the field, and thus the scheme of the database is detailed and well-documented. However, since the information populating the database is provided by third parties, its quality varies greatly (14).

### 3.1. Data description

**Figure 1:**
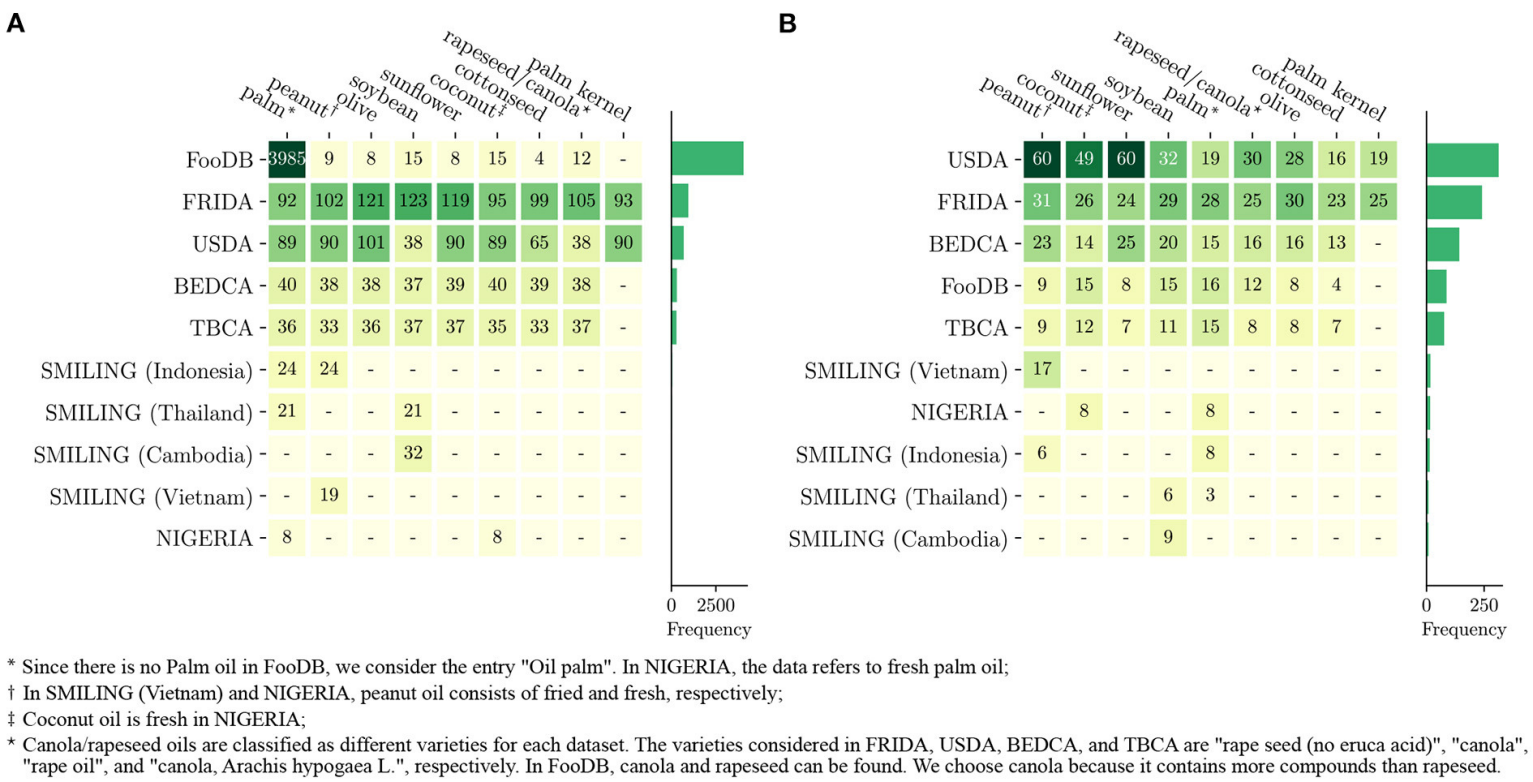
Number of compounds and nutrients reported for each vegetable oil in the selected databases (EuroFIR not included). **(A)** The total number of nutrients for which the database provides some information, while **(B)** contains only those nutrients with a quantity larger than 0. In both panels, the bar plots represent the sum of the values of the same line, and the rows and columns of the matrix are ordered in decreasing order from left to right and from top to bottom.

For this analysis, we focus on the major vegetable oils in terms of world supply and distribution: coconut, cottonseed, olive, palm, palm kernel, peanut, rapeseed/canola, soybean, and sunflower oils (74). Figure 1 illustrates the amount of information on these vegetable oils contained in the selected databases. In Figure 1A we report the total number of compounds present in each database. According to this, FRIDA is the database with the largest amount of information, superseding the USDA database, except for palm oil, for which FooDB provides an enormous amount of compounds. In terms of the overall coverage of each oil, palm oil is the most studied one, followed by peanut oil and olive oil. However, many of the entries in these databases are 0 (the distinction between measured 0 and logical 0 is seldom made). If those nutrients are removed, the depicted scenario changes completely Figure 1B.

Indeed, if we focus only on those nutrients with a reported presence larger than 0, the USDA turns out to be the database with the largest amount of information. Besides, the most studied oil is peanut oil, followed by coconut and sunflower oils, while palm and olive oil move to the 5th and 7th positions, respectively. In FRIDA, the number of nutrients with a quantity larger than 0 is one-third of the total amount of nutrients studied, contrasting with the USDA database in which only half of the nutrients are quantified as 0. This depicts a very different scenario in terms of micronutrients present in vegetable oils depending on the database analyzed.

The rest of the databases contain much less information than the first two. The smallest ones (NIGERIA and SMILING) focus specifically on regional foods, and thus it is expected that these datasets do not report much information on vegetable oils that are not common in these countries. It is also interesting to note that while FooDB contains information about thousands of chemical compounds, the quality of said information is relatively low since the actual amount of quantified compounds per vegetable oil is even lower than in the FCDBs that it uses as source. The low quality of the metadata—if present—is also a major problem, as it is usually impossible to know the analytical procedure used, the cultivar, variety or simply the species of the element.

Lastly, we must note that we have not included EuroFIR due to the heterogeneity of its data. Currently, the database has a set of guidelines that contributors have to follow when uploading information to the system, but that does not guarantee that they will follow them, nor that the original information has sufficient quality (27). For instance, the number of countries reporting information is quite variable: 36 for olive oil; 30 for sunflower oil; 22 for palm oil; 22 for coconut oil; 21 for peanut oil; 13 for soybean oil; and 9 for cottonseed oil (note that EuroFIR now includes some non-European countries). Regarding the quality of the documentation, even though EuroFIR requires information on the analytical method used to measure the composition, in most cases, it is reported as “unknown.” Similarly, it is mandatory to provide the source of the data, but in many cases, it is either not reported or not well-described (e.g., “No change from USDA”). Even though the platform is a huge step forward in the right direction, there are still many values that are not fully comparable (14). Solving these issues is beyond the scope of our paper, and thus we have not included it in the subsequent analysis.

### 3.2. Qualitative comparison

Next, we look at the age of the information to evaluate the validity of the data. From a broader perspective, the problem of outdated information can be related to the issue of data obsolescence. Obsolescence refers to the appearance of a new piece of information that supersedes an existing one that is still available (75). Some authors propose the use of machine-learning techniques to detect when data becomes obsolete or contradicts previous knowledge (76). In our context, as previously discussed, the composition of food is continuously changing as a consequence of both natural and human interventions. Besides, analytical techniques keep improving, giving more detailed and precise estimations. As such, it is important to both keep the databases updated and, at the same time, store the old information so that dietary studies carried out in the past can use the proper composition.

From the databases analyzed, only USDA, BEDCA, FRIDA, and TBCA provide detailed information on the year when the content was measured. In the subsequent analysis, for USDA, we considered only SR Legacy, when available, so as to be able to analyze the dates of all compounds separately. Yet, note that when the data are extracted from a scientific publication, the date that is associated is the one when it was published, not when the product was actually analyzed. Thus, unless it comes from direct estimation, any value might have been measured at the depicted date or before. In fact, many compounds share the same date, but that is because they were extracted from a compilation or a database published in that year and not because they were measured in that year.

**Figure 2:**
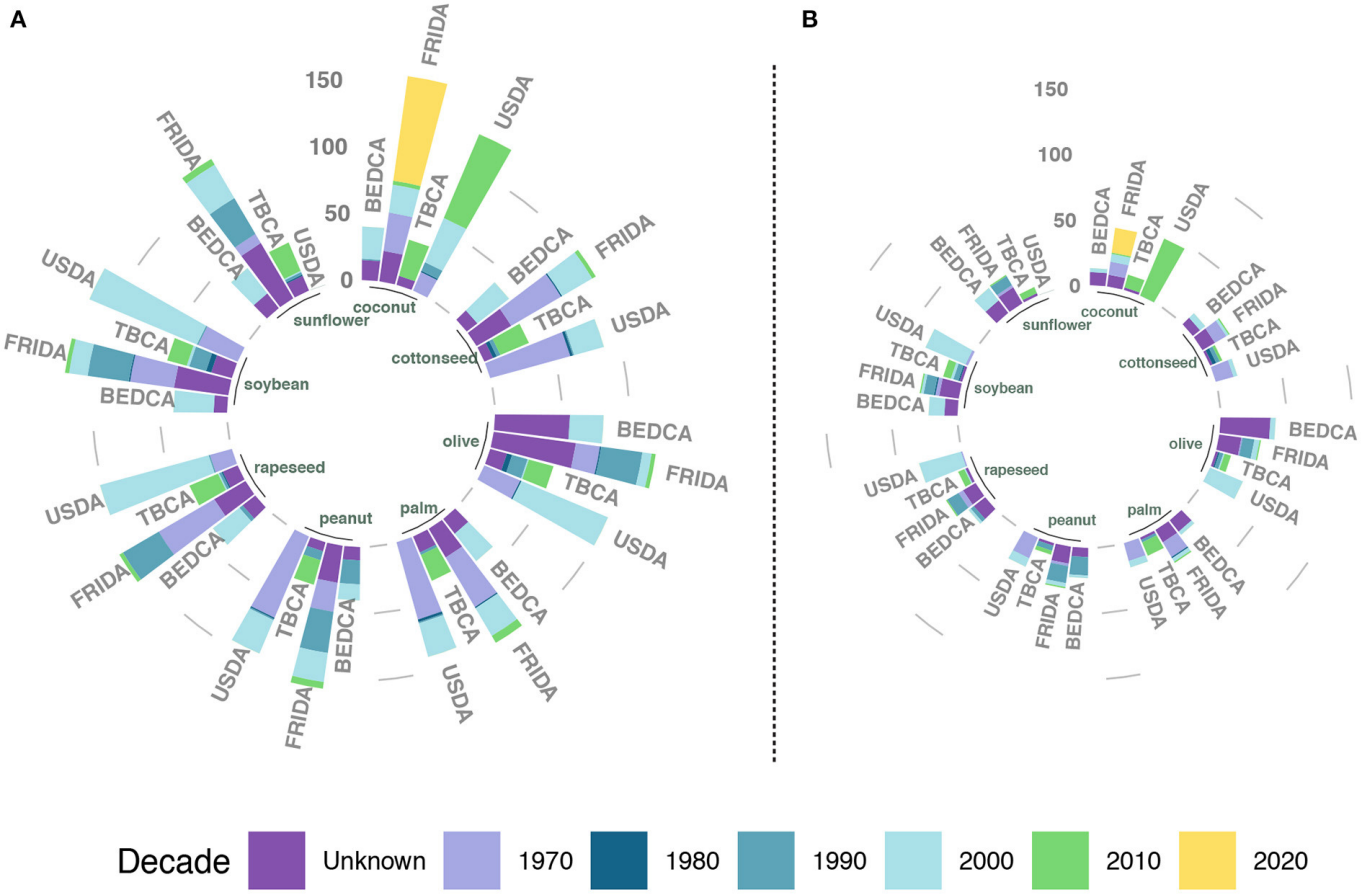
Date associated with each compound or nutrient in the selected databases that provide said information. For each vegetable oil, the length of the bar indicates the number of compounds, while the color represents the amount of them that have an associated date within a decade. Palm kernel is not included as it is only reported in FRIDA and USDA. **(A)** The information for all elements present in the database, regardless of their actual value, while **(B)** shows the information only for those with a reported quantity larger than 0.

Figure 2 shows the number of compounds classified by the decade corresponding to the listed year in their source. As we can see, the information tends to be decades old, questioning its validity. The selected USDA database does not contain any information collected after 2010, except for coconut oil which was substantially updated in 2015. Similarly, all the information contained in BEDCA comes from the decade of 2000. A closer inspection reveals that most information comes from either a book published in 2004 or from the USDA database that was available back then. Yet, as we can see, even though they used the version that was available at the time, the information contained there could already be decades old. For the FRIDA database, we observe that most of the information comes from three different dates separated by a decade, signaling that the speed of the updates is relatively low. Lastly, TBCA is the most updated one, which is to be expected since it started in the decade of 2010 and most of the information comes from direct analyses.

Yet, a closer inspection reveals more weaknesses. For instance, focusing on the case of palm oil, we observe that in the USDA database most of the information comes either from 1979 or from the early 2000s, with the last update in 2009 (folate). It is important to note that the values which are assumed to be 0 are usually not updated, explaining why there are so many compounds that have not been updated since 1979 in Figure 2A. Note also that, as previously discussed, a value of 0 might mean different things in each database: below detection limits, not analyzed, assumed to be 0, etc. In Figure 2B, when we remove those elements whose concentration is reported as 0, we observe an important reduction of compounds with information from that period. A similar result was found for FRIDA, with the majority of the data also included in two dates. In the case of TBCA, even though the source is supposed to be recent, there are several compounds whose information was extracted from the USDA in 2017. Given that the USDA has not updated the information on palm oil since 2009, the information contained in TBCA is actually a decade older than reported. All in all, if we consider USDA, FRIDA and BEDCA, 57.9% of the information was collected before 1990 and the remaining before 2010.

### 3.3. Quantitative comparison

**Figure 3:**
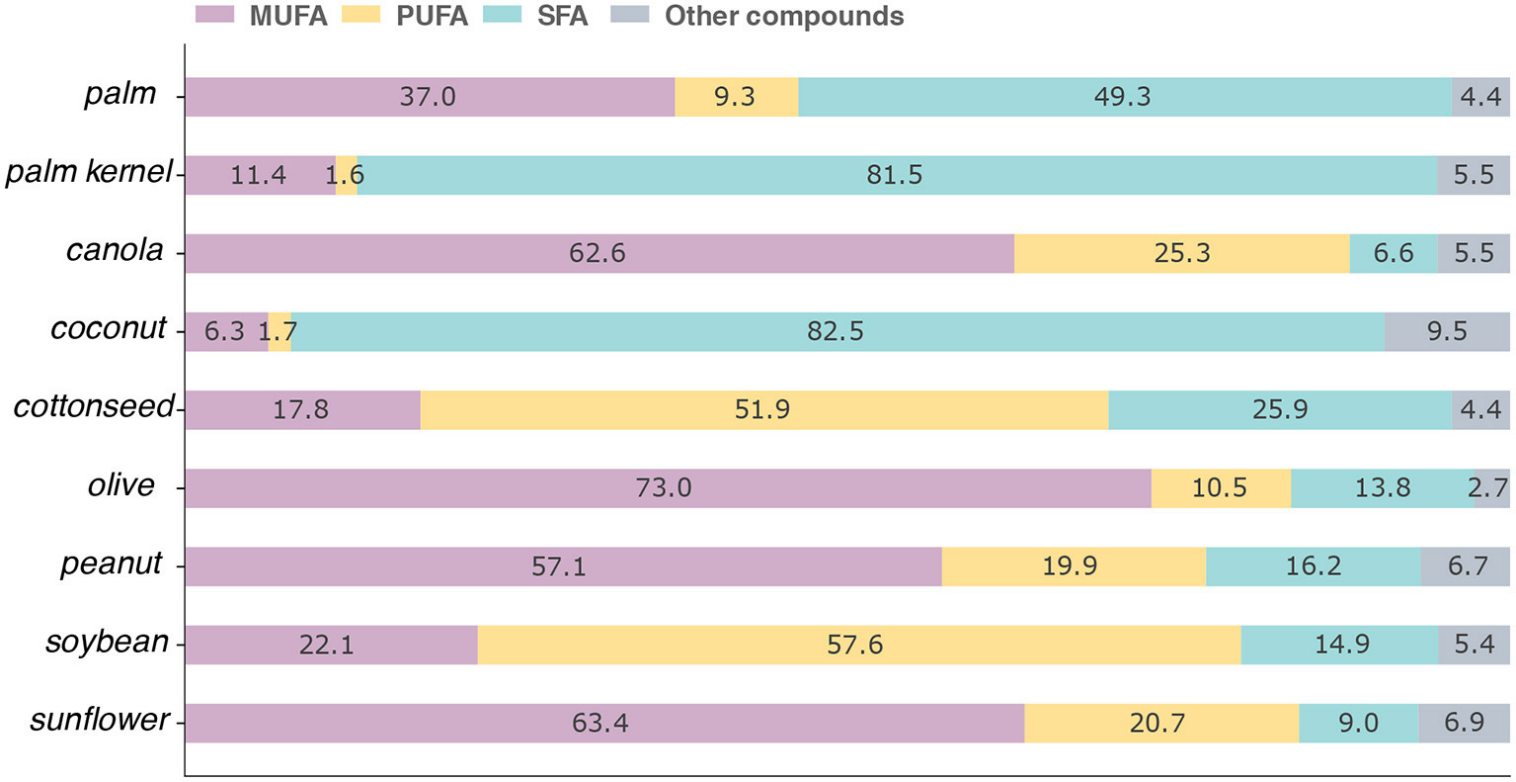
Comparison of fatty acids among different oils in the USDA database. The values are shown as the percentage out of 100 g of the product. MUFA, PUFA, and SFA represent monounsaturated, polyunsaturated, and saturated fatty acids, respectively. Other compounds denote elements that were not described in the database and that would be necessary to reach 100%.

In Figure 3 we show the fatty acid composition of the oils contained in the USDA. Specifically, canola, coconut, peanut, soybean, and sunflower oils are from Foundation foods, palm, palm kernel, and olive oils are from SR Legacy, and cottonseed oil is from Survey Foods (FNDDS). As expected, each vegetable oil has a very different composition, which highlights why it is so important to have precise information about as many foods as possible. If a product is substituted simply with one that seems similar, one may incur in important errors when estimating the actual intake. Having extensive documentation is also very important to understand the information. For instance, common sunflower oil usually has a concentration of 20% monounsaturated fatty acids, which contrasts with the 60% reported in the USDA. A closer inspection of this database shows that the value is the average of eight samples, two of which have about 20% of MUFA and six with around 75–80%. In other words, they are averaging the composition of two samples of common sunflower oil and 6 samples of high oleic sunflower oil. If the information on individual samples is not available, it is impossible to understand the origin of certain discrepancies, and any estimation done with these values might be biased.

**Figure 4:**
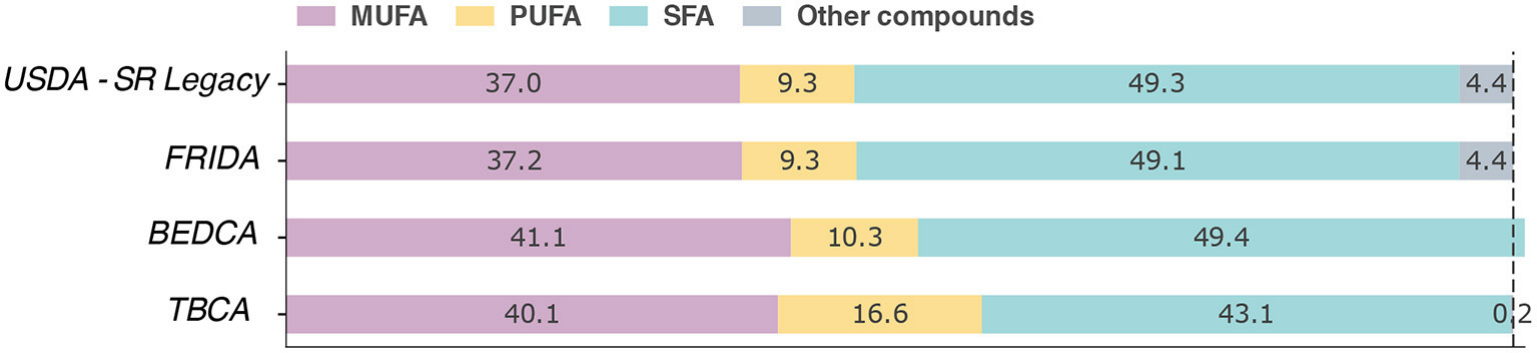
Comparison of fatty acids in palm oil among different FCDBs. The values are shown as the percentage out of 100 g of the product. MUFA, PUFA, and SFA represent monounsaturated, polyunsaturated, and saturated fatty acids, respectively. Other compounds denote elements that were not described in the database and that would be necessary to reach 100%.

Following the previous example of palm oil, we now look at the composition in terms of fatty acids in the databases explored in the previous section (Figure 4). It is worth noting that for BEDCA the sum of all fatty acids is 100.94 g per 100 g, a common inconsistency found in FCDBs that extract their information from a combination of scientific publications. Furthermore, the only components that are not 0 are fatty acids and alpha-tocopherol. In contrast, the sum of all fatty acids in the USDA and Frida databases is 95.4 g per 100 g, and they also report the presence of vitamin K.

At first glance, all databases share similar values. However, upon closer inspection, one can see that the amount of polyunsaturated fatty acids reported by TBCA is 78% higher than the one in USDA and FRIDA. As previously discussed, there are many factors that can alter the composition of a product. Regarding the source material, different species will have different nutrient contents, and even within the same species the composition may change substantially from cultivar to cultivar (2). Besides, the particular season when it was harvested, or the production processes can also alter the composition. Thus, the main concern is not that the values are different but that there is no information in the databases that allows one to determine what could be their cause. One solution to this problem would be to include additional metadata with information on species, variety, cultivar, etc.

Another example is the reported concentration of palmitic acid. A study from 1973 showed that samples from Zaire, Indonesia, and Malaysia contained, on average, 42, 48.6, and 49.2 g, respectively (77). In contrast, in the FCDBs considered the concentrations are much closer to one another: 43.50 g for USDA; 43.68 g for FRIDA; and 43.04 g for BEDCA (TBCA does not report the quantity of this fatty acid). This may be caused by the importation of palm oil from the same area, which would explain the similarities. However, it signals that the values might be ill-suited for countries that may not obtain it from the same source. Lastly, even though it was not included directly in the analysis, if we look at the value reported by FooDB we get that the median concentration is 25.8 g, wildly different than in any other database. Fortunately, it is possible to download the raw information, which reveals that the website is averaging the values of both palm oil and palm kernel oil, even though the latter has a completely different composition.

## 4. The challenges for a Big Data approach

As we have seen, FCDBs collect a lot of information from scientific publications, and they may lose very valuable information in the process. Besides, they also tend to neglect biodiversity and the temporal and spatial dimensions of food composition, weakening the conclusions that can be reached using that data. One possibility to update and enrich the quality of FCDBs would be to systematically review the literature and extract as much information as possible, which can then be studied using Big Data techniques—a task full of challenges.

Continuing with the example of palm oil, we can estimate the number of scientific records that are relevant for this purpose using the information of scientific records from Microsoft Academic Graph (MAG) (78). In particular, we used the version that contains publications up to 25th of June 2020, provided by the CADRE project from Indiana University (79). Considering the abstracts and titles, we recovered all entries with the words “palm” or “elaeis” and “oil.” As a result, we obtained 79,210 documents. Taking this information, we created a network of citations between these documents. Specifically, each document (e.g., a paper or a book) represents a node in the network, and two documents are linked if they reference each other. This allows us to classify documents according to their content since papers that belong to the same subfield tend to cite each other more. After removing the nodes that are disconnected from the rest of the network (they have no citations with any other documents of this subset of scientific records), we end up with a network of 29,912 nodes. Next, we extract the communities from this network (80, 81). In network science, communities are groups of nodes which have much more connections between each other than expected. Furthermore, we automatically assign them descriptive labels using a topic modeling technique (82, 83).

**Figure 5:**
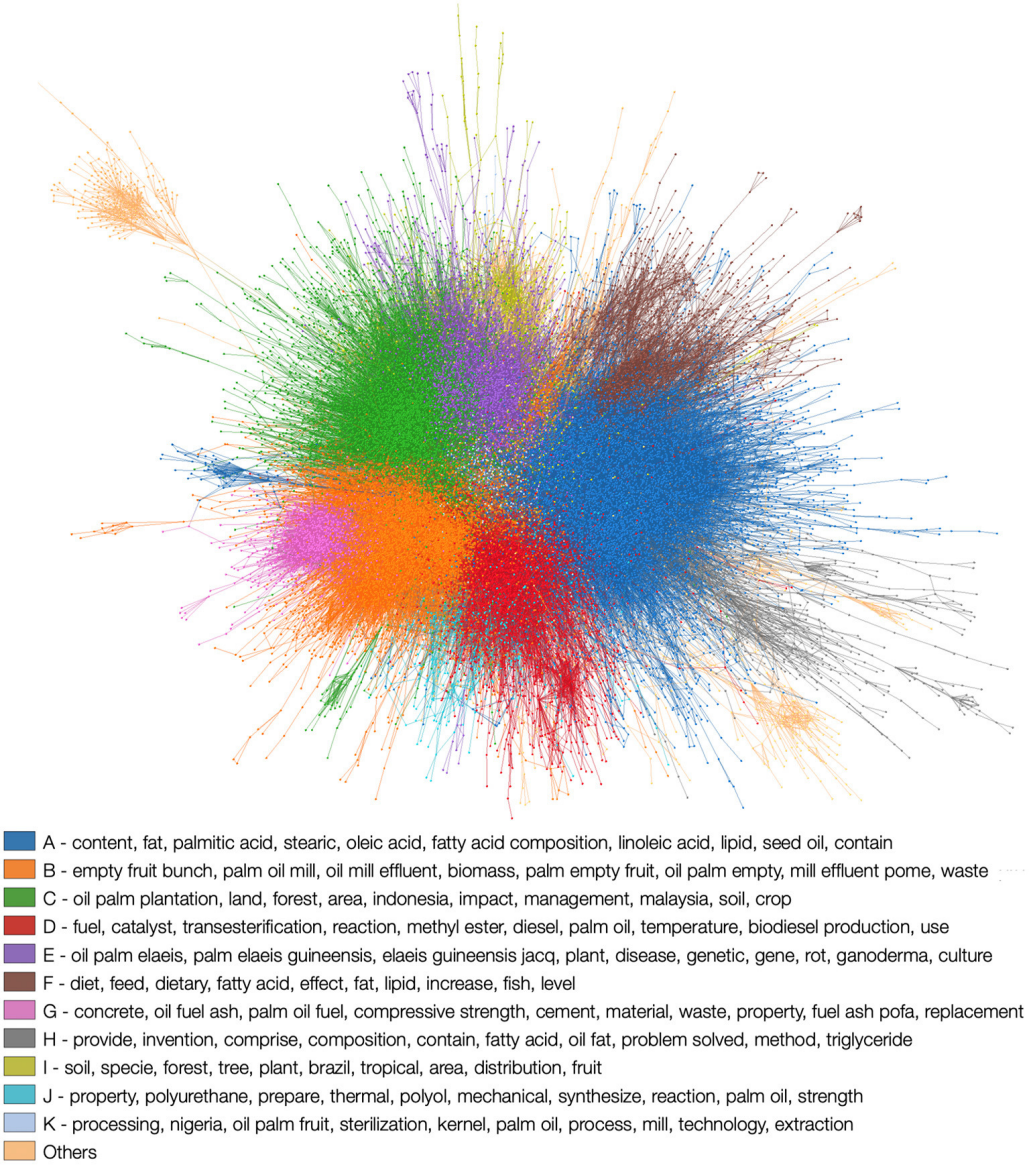
Visualization of the palm oil citation network. Scientific records that cite each other can form communities, signaling that they contain similar information. Each color represents a community detected in the network and the labels are the keywords that determine the contents present in the community. Communities are ordered according to their size, with A being the largest. This network was plotted using the software implemented in Silva et al. (82).

In Figure 5, we depict the communities obtained, ordered in decreasing order according to their sizes. The keywords within each community are ordered in decreasing order for each community according to their importance. We define importance as the difference between the normalized frequencies of *n*-grams of a given community, and the normalized frequencies of *n*-grams excluding it (an *n*-gram is a set of *n* consecutive words in a text) (83). We can see clearly that the keywords of community A—the largest one—are related to food composition. In the case of community B, it seems to be related to the processing and resulting waste. Community C keywords can be related to the regions and plantations. In D, the keywords are related to applications for palm oil, such as its use for producing biodiesel, etc. Thus, one could focus on studying the 2,293 publications belonging to community A.

The next step would require the application of advanced text mining techniques that could extract the information contained in the papers (84, 85). However, the unstructured nature of these publications makes this a very complex task (86). Furthermore, it is not clear if the results would be valuable enough. One of the problems of Big Data is the high dimensionality of the information, which brings noise accumulation and may introduce spurious correlations. If the information is of low quality, increasing the amount of papers will only exacerbate these issues. Besides, aggregating information from so many different sources will inevitably mix results obtained in different locations, times and with different technologies, which introduces further systematic biases and quality issues (87). As such, simply extracting the pair nutrient - quantity is not enough. Instead, it is necessary also to determine exactly how the sample was analyzed, its specific variety, when and where it was harvested/produced, etc. Not only this represents a much harder task, but it also may not be achievable since much of this metadata might not be contained in the own publication in the first place (88).

Among the AI approaches, Natural Language Processing (NLP) techniques (89) are particularly useful because they can help extract information from the scientific literature. Additionally, recommendation systems have been explicitly created to retrieve and filter scientific papers, which can combine information of different natures (e.g., citation network and paper content) (90). With a set of documents adequately selected, it may be easier for specialists to extract and validate data from the literature. However, one can also automatically look into the content of the papers using NLP. Many techniques have been used to extract and represent the semantics of the texts. Some successfully used methods are the embeddings (91–93), such as *word2vec* (92) and *doc2vec* (93), which represent words and documents, respectively. More recently, transformers were proposed (94). Among the most successful ones are the Bidirectional Encoder Representations from Transformers (BERT) (95) and the Generative Pre-trained Transformer 3 (GPT-3) (96), which can be used as part of systems devoted to retrieving information from documents of different domains (97–101). As such, developing and extending these approaches to assist in constructing better FCDBs is a promising area of research that could help to improve our knowledge about food, nutrition and health (42).

## 5. Conclusion

The decade of 1980 kicked off a global effort to homogenize and standardize the way in which nutritional composition is collected in order to make meaningful comparisons between countries. Since then, initiatives like INFOODS or EuroFIR have established very clear guidelines and best practices that should be followed to properly obtain, document, store and share this type of data. However, many times, probably due to a lack of funding, these guidelines are not fully adhered to. Furthermore, the end users of these data are usually not fully aware of its limitations and many times complement it with information extracted from sources that are not totally compatible. This may lead to wrong conclusions and misguided policies, with impacts that can take years to fix.

In the age of data, it is more important than ever to ensure that it is correctly captured and displayed. In this contribution, we have discussed that most FCDBs already have many problems with the little information they report. In the particular case of vegetable oils, we have demonstrated that missing information is not always handled properly, that many sources commonly used are old (see Supplementary Table 1) or have mistakes in them (assuming that the source is provided, which is not always the case), and that the quantitative composition can either vary a lot or not at all, without knowing the reasons behind that. This problem is also present in the global scientific literature, not only in FCDBs, which hinders the possibility of reaching precision nutrition. Initiatives such as Foundation foods from USDA are heading in the right direction, but the effort should be much more global and, importantly, sustained in time.

In terms of FCDBs and artificial intelligence (AI), there are two crucial points. The first issue is the urge for good quality data to train AI models properly, and the second is how AI can help feed these databases. Both aspects are related and interdependent because without having data, it is challenging to train models, and without good models, it is much more demanding to enhance the databases. In this paper, we have shown a perspective on the amount of scientific data that has to be processed to extract information regarding a single food item, palm oil, if one wants to scan the information already present in the literature. If this is to be done for many food items, the volume and challenges will increase even further. Nonetheless, we expect that the development of AI in food-related research can positively impact the overall quality of FCDBs, as it has done in other areas of nutrition (102, 103).

There will be many new challenges in this process. This type of analysis will require the collaboration of researchers from different knowledge areas, including network science, neural language processing, food chemistry or nutrition. For the development of new machine learning approaches, it will be essential to include experts in food composition data to evaluate the quality of the information and guarantee the overall quality of the database. As noted, this is a complex task, and the problems related to FCDBs can only be mitigated if experts in many areas put their efforts together. A related problem is the necessity of new funding opportunities for interdisciplinary research projects. Even though large funding agencies actively encourage proposals that cross disciplinary boundaries, in practice most funded projects remain firmly in a disciplinary framework (104).

To conclude, nowadays, the sustainability of the food system is being questioned in the pursuit of the SDGs. Food production is closely related to public health and the environment, and proper knowledge of what we eat is key to improve both. The lack of information on many aspects, such as food fortification or biodiversity is inevitably hindering the progress toward a better food system. Besides, climate change is already having a measurable effect on crops, and not only it will increase in the future, but as we adapt to it, our consumption patterns might change. To mitigate possible further nutritional problems and to solve the ones that we already have, gathering and curating much more and better data is imperative. As the food sector digitizes, it is essential to acknowledge the importance of pursuing a holistic view of nutrition and to move toward a data-driven food system. Only then relevant players will be able to issue evidence-based and timely policy recommendations.

## Author contributions

HF, AA, and YM: conceptualization, methodology, investigation, writing–original draft preparation, and writing–review and editing. HF: software, validation, and data curation. HF and AA: visualization. All authors have read and agreed to the published version of the manuscript.
