# Supplementary material for: Food composition databases in the era of Big Data: Vegetable oils as a case study
Source: Front Nutr. 2023 Jan 5;9:1052934. doi: 10.3389/fnut.2022.1052934 (PMC9851468; doi:10.3389/fnut.2022.1052934)
Supplement: Supplementary file 1 [file Data_Sheet_1.pdf]

SUPPLEMENTARY TABLE

Database	Oil	Uknown	1970	1980	1990	2000	2010	2020
BEDCA	coconut	38	-	-	3	60	-	-
	cottonseed	25	-	-	-	75	-	-
	olive	69	-	-	-	31	-	-
	palm	35	-	-	-	65	-	-
	peanut	25	-	-	45	30	-	-
	rapeseed	30	-	-	8	63	-	-
	soybean	25	-	-	-	75	-	-
	sunflower	37	-	-	-	63	-	-
FRIDA	coconut	15	19	-	-	14	2	51
	cottonseed	29	42	1	-	25	3	-
	olive	51	15	1	25	6	2	-
	palm	25	43	1	-	24	6	-
	peanut	26	20	-	29	20	5	-
	rapeseed	25	44	-	28	-	3	-
	soybean	33	27	1	25	11	2	-
	sunflower	40	7	-	26	24	4	-
TBCA	coconut	20	-	-	-	-	80	-
	cottonseed	19	-	8	8	-	64	-
	olive	27	-	8	24	2	39	-
	palm	36	-	-	6	-	58	-
	peanut	21	-	-	21	-	58	-
	rapeseed	31	-	3	5	3	59	-
	soybean	32	-	8	26	4	30	-
	sunflower	31	-	3	5	3	59	-
	coconut	-	12	1	6	28	54	-
USDA	cottonseed	-	70	2	1	26	-	-
	olive	-	29	1	-	70	-	-
	palm	-	67	2	1	29	-	-
	peanut	-	69	1	1	28	-	-
	rapeseed	-	17	-	1	82	-	-
	soybean	-	27	-	1	73	-	-

Table 1. Age of the information. Each value represents the percentage of the nutritional information contained in each oil whose source is associated to a given decade. Note that even though the source of the information might have a certain date associated (e.g. when it was published) that does not mean that the information was originally measured on that year.
